# Cellulose Diacetate Aerogels with Low Drying Shrinkage, High-Efficient Thermal Insulation, and Superior Mechanical Strength

**DOI:** 10.3390/gels10030210

**Published:** 2024-03-21

**Authors:** Sizhao Zhang, Kunming Lu, Yangbiao Hu, Guangyu Xu, Jing Wang, Yanrong Liao, Shuai Yu

**Affiliations:** 1 Polymer Aerogels Research Center, Jiangxi University of Science and Technology, Nanchang 330013, China; 2 Science and Technology on Advanced Ceramic Fibers and Composites Laboratory, National University of Defense Technology, Changsha 410073, China; 3 Postdoctoral Research Station on Mechanics, College of Aerospace Science and Engineering, National University of Defense Technology, Changsha 410073, China

**Keywords:** cellulose, aerogel, drying shrinkage, thermal insulation, mechanical strength

## Abstract

The inherent characteristics of cellulose-derived aerogels, such as their natural abundance and environmental friendliness, make them highly interesting. However, its significant shrinkage before and after the supercritical drying procedure and low mechanical strength limit its potential application. Here, we propose a strategy to prepare cellulose diacetate aerogels (CDAAs) with low drying shrinkage, exceptional thermal insulation, and superior mechanical strength. The low drying shrinkage (radial drying shrinkage of 1.4%) of CDAAs is attributed to their relative strong networking skeletons, which are greatly formed by tert-butanol solvent exchange in exerting the interaction of reducing the surface tension force. In this case, CDAAs are eventually endowed with the low bulk density of 0.069 g cm^−3^ as well. Additionally, as-prepared CDAAs possess an abundant three-dimensional networking structure whose pore size is concentrated in the diameter range of ~50 nm, and the result above is beneficial for improving the thermal insulation performance (thermal conductivity of 0.021 W m^−1^ K^−1^ at ambient environmental and pressure conditions). On the other hand, the optimal compressive stresses of CDAAs at 3% and 5% strain are 0.22 and 0.27 MPa respectively, indicating a mechanically well robustness. The above evidence demonstrates indeed the exceptional thermal insulation and superior compressive properties of CDAAs. This work may provide a new solution for developing a kind of high-performance cellulose-derived aerogel in the future.

## 1. Introduction

The most common natural polymer is cellulose in nature [1,2]. In the trend of green chemistry [2], cellulose stands out among many polymers due to its widely available resources, low cost, and degradability. This makes it an environmentally friendly choice for various applications, contributing to sustainable and eco-friendly practices in material science and industry [3,4,5]. Cellulose-derived aerogels are mainly composed of cellulose with environmentally friendly properties [6], which possess a series of excellent characteristics such as easy surface modification, high porosity, high specific surface area, low density, and 3D interconnection [7,8]. Combined with the exceptional performances of an aerogel [9], cellulose-derived aerogels are also renewable and biodegradable, so they are called “the third-generation aerogel” [10,11,12,13]. In the construction sector, the growth of housing construction and the trend towards higher thermal comfort levels for humans have brought about an increase in energy demand, which has a negative impact on the environment [14]. With the growing demand for renewable insulation in green and energy-efficient buildings, cellulose-derived aerogels are becoming the next generation of thermal insulation, which is biodegradable, inexpensive, and sustainable. This biomass-based aerogel material helps to prevent the related heat loss inside the building, further reducing the practical need for heating and cooling systems. In cold or hot climates, it is beneficial to reduce the dependence on air conditioning systems to a great extent, which can significantly decline the energy consumption of buildings. Furthermore, the aerogels above have outstanding shock and sound absorption abilities, which enhance the seismic capacity of buildings in a way and decrease the noise level both indoors and outdoors. The superior properties of cellulose-derived aerogels can be utilized to create the thermal insulators for the energy-saving of buildings (characteristics: environmentally friendly and sustainable) [15,16].

Generally, cellulose-derived aerogels can be broadly classified into three categories according to the source and structure of cellulose preparation [17,18,19], namely nanocellulose aerogels, regenerated cellulose aerogels, and cellulose derivative aerogels [20]. One way to achieve a nanocellulose aerogel is by dissolving and dispersing nanoscale cellulose, which is crosslinked to form a hydrogel and then dried [21]. Conventional regenerated cellulose aerogels have a larger average pore size compared to nanocellulose aerogels, which are obtained from a series of chemical treatments [22]. In contrast, cellulose-derived (namely cellulose derivatives) aerogels could be chemically modified and regulated due to the surface modification of cellulose, which accordingly has the ability to enhance and improve the original properties of cellulose-derived aerogels despite their different needs [23,24]. Currently, the chemical modification of cellulose mainly includes oxidation, esterification, etherification, and other methods. This chemical treatment mainly used amino, epoxy, carboxyl, or aldehyde groups to react with hydroxyl groups, to finally obtain sufficient structural stability. To sum up, cellulose-derived aerogels are considered to be the most valuable biomass-based aerogels (originating from a cellulose resource) owing to the widely easy modulation and the moderate reactive activity from a chemical-structure perspective. However, to date, the mechanical strength of cellulose-derived aerogels is always challenging, necessitating further research to increase their performance [25,26,27].

To address the issue above, Peng et al. [21] used a bionic hybrid strategy to prepare an effective multistage ordered high-strength insulating nanocellulose-based aerogel. The cellulose-based aerogel was dried at room temperature and hot-pressed to create a stronger H-bonded crosslinked network. It exhibits excellent mechanical properties and a low thermal conductivity of 0.021 W m^−1^ K^−1^. Yao et al. proposed a simple method to crosslink cellulose aerogels during solvent exchange using the Schiff base reactions. The detailed procedures are presented as follows. First, a silane coupling agent of 3-aminopropyltriethoxysilane (APTES) was added to a bacterial cellulose (BC) dispersion. APTES was hydrolyzed to form polysiloxanes, which were attached to the surface of BC fibers during the gelation process. Then, the BC gel was immersed in an ethanol solution containing glutaraldehyde for solvent exchange and crosslinking by a crosslinking technique. On the other hand, the hydrolyzed APTES encapsulated on BC can enhance the mechanical properties of single fibers by self-crosslinking. Specifically, APTES could build the amine-rich structures on the surface of BC, which promotes the formation of the inter-fibers by Schiff base reactions. By virtue of the double crosslinked networks, the gel backbone of BC nanofibers can be significantly enhanced to withstand the capillary forces during the evaporation of solvents such as ethanol. The mechanical strength of the cellulose-based aerogel obtained could be improved by means of the physical and chemical introduction of APTES from a macroscopic-strength perspective. However, the low drying shrinkage of a cellulose-based aerogel always becomes obvious because of the enhancement of networking skeletons, and even causing the poor thermal insulation property. Hence, how to synthesize cellulose diacetate aerogels with low drying shrinkage, high-efficient thermal insulation, and superior mechanical strength is an urgent problem that needs to be solved.

On the basis of our previous work [28,29], we proposed a facile preparation method to construct a kind of high-performance cellulose acetate aerogel (CDAA) by the crosslinking of cellulose diacetate and 2, 4-toluene diisocyanate, which experiences the aging process, a tert-butanol solvent exchange, and finally supercritical carbon dioxide. The microstructure, drying shrinkage, thermal insulation, and mechanical properties of CDAAs were systematically investigated. Benefiting from the introduction of tert-butanol, the CDAAs retained their porous structure during solvent removal under the interaction of tert-butanol solvent polarity, which resulted in a minimal drying shrinkage (1.4%) of CDAAs before and after the drying process compared with those of the other studies [28,29,30]. The thermal conductivity of 0.021 W m^−1^ K^−1^ at ambient temperature and pressure suggested the presence of moderate nanoporous structures and the corresponding excellent thermal insulation property of CDAAs. In addition, the well as-prepared aerogels had a more consistent and denser internal network of CDAAs, which further improved the skeleton strength from a micro-scale view. This research may serve as a new reference with respect to cellulose-derived aerogels in the thermal-insulators domain of energy-saving buildings in the future.

## 2. Results and Discussion

### 2.1. Macrostructure and Microstructural Evolution

**Figure 1:**
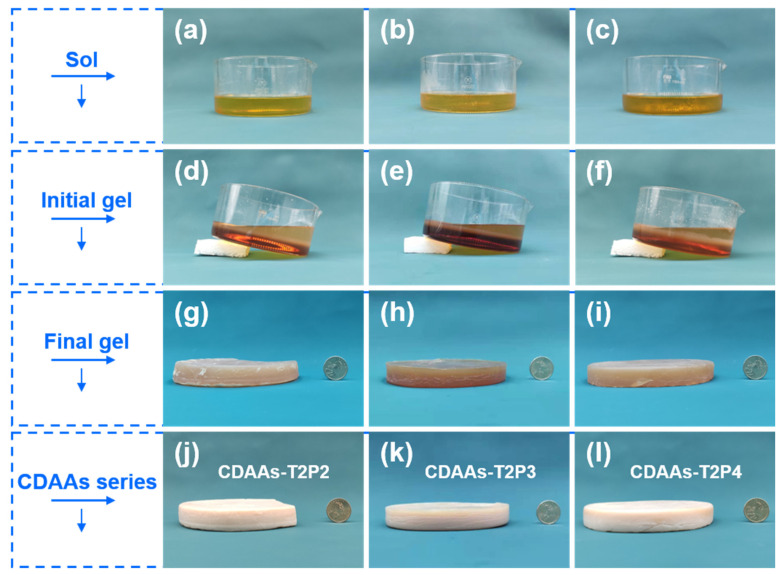
Photographs of the corresponding sol, initial gel, final gel, and aerogel in the CDAAs’ preparation processes: the respective stages for (**a**,**d**,**g**,**j**) CDAAs-T2P2, (**b**,**e**,**h**,**k**) CDAAs-T2P3, and (**c**,**f**,**i**,**l**) CDAAs-T2P4.

**Figure 2:**
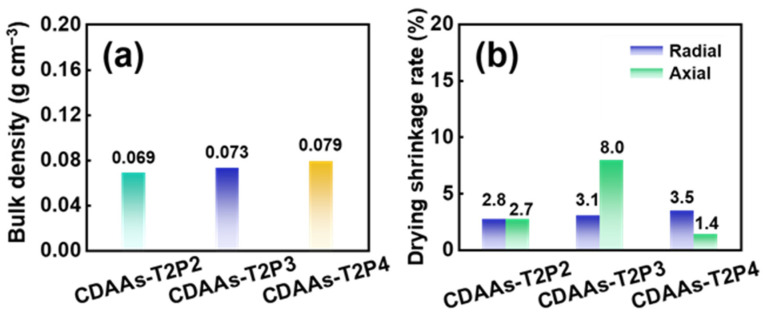
Bulk density (**a**) and drying shrinkage (**b**) of CDAAs prepared with different catalyst contents.

Figure 1 indicates photographs of the corresponding sols, initial gels, final gels, and aerogels during the preparation process of CDAAs. CDAAs-T2P2, CDAAs-T2P3, and CDAAs-T2P4 are generally well-shaped and beige. As in Figure 1, the height of the aerogel is less than the size of an upright coin. The final gels and aerogels exhibit no appreciable variation in physical appearance across radial and axial directions, implying a generally uniform size and the relatively small drying shrinkage (Figure 2b). Figure 2 shows a comparison of CDAAs-T2P2, CDAAs-T2P3, and CDAAs-T2P4’s density and drying shrinkage. The bulk densities of CDAAs-T2P2, CDAAs-T2P3, and CDAAs-T2P4 correspond to 0.069, 0.073, and 0.079 g cm^−3^ respectively, which indirectly indicate that a pyridine catalyst has an effect on the degree of crosslinking. On the other hand, the axial shrinkage of CDAAs-T2P4 is as low as 1.4%, signifying the very excellent inhibition of drying shrinkage before and after the supercritical drying process by this preparation method and TAB as exchange solvent in this work. This behavior can be linked to the fact that the TAB solvent effectively decreases the intermolecular forces caused by solvent evaporation and further avoids the damage to the gel networking skeleton structure during the solvent replacement process.

**Figure 3:**
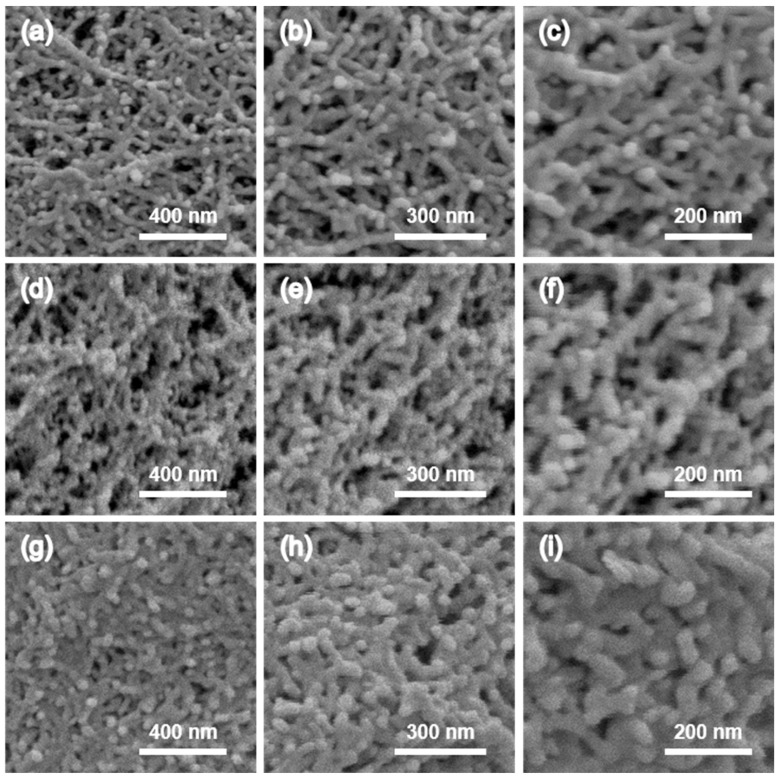
FESEM images of CDAAs showing the textural structure at different magnifications: (**a**–**c**) CDAAs-T2P2, (**d**–**f**) CDAAs-T2P3, (**g**–**i**) CDAAs-T2P4.

As shown in Figure 3, the cross-sectional surface morphology of CDAAs shows a nanoscale networking skeletons in all aerogels as prepared in a comprehensively typical view. To obtain the fine crosslinked structures of CDAAs, the microstructures of CDAAs were successively magnified. FESEM observation revealed dense networking structures in CDAAs-T2P2, with pore diameters predominantly within the range of ~50 nm. In the condition of a relatively low catalyst dosage, the porous structure of CDAAs may become fine, forming the loose networking skeletons and the low bulk density, further lowering their compressive strength. Conversely, at a high catalyst dosage, the porous networking structure of CDAAs may shrink due to the further crosslinking reactions, resulting in an increase in their bulk density and a decrease in their pore size accordingly, meanwhile increasing their compressive strength. In more detail, an increase in pyridine dosage results in a bigger cross-sectional diameter and smaller pore size for the networks of CDAAs-T2P3 compared with that of CDAAs-T2P3. This phenomenon may be the outcome of the random expansion of networking skeletons, eventually leading to the corresponding smaller pores in Figure 3. The morphological texture of CDAAs-T2P4 is achieved with a denser network structure compared to the other two groups above. Meanwhile, the pore size of CDAAs-T2P4 is also regulated, obtaining the relatively small pore structure. In summary, CDAAs-T2P4 acquires the excellent mechanical properties, simultaneously maintaining a good thermal insulating property (Table S1). As can be seen from the thermal conductivity data, CDAAs-T2P3 has the lowest thermal conductivity (as low as 0.021 W m^−1^ K^−1^) in all the groups.

**Figure 4:**
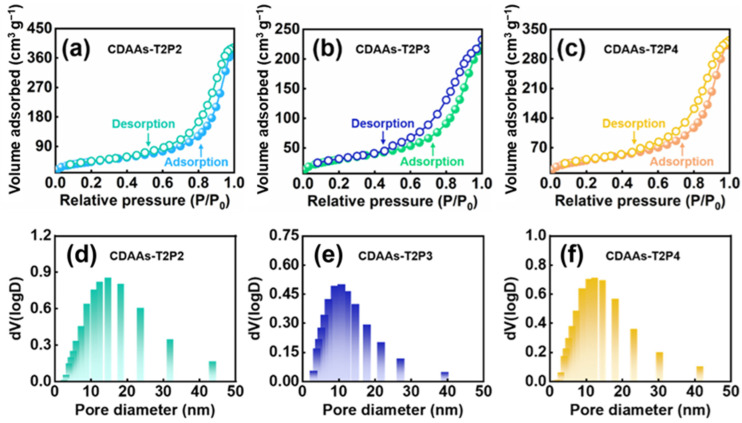
N_2_ adsorption–desorption isotherms and pore distributions of CDAAs: (**a**,**d**) CDAAs-T2P2, (**b**,**e**) CDAAs-T2P3, (**c**,**f**) CDAAs-T2P4.

The pore structure of CDAAs can be characterized by a nitrogen absorption and desorption analyzer. Figure 4 illustrates the nitrogen adsorption–desorption characteristics and pore size distributions of CDAAs-T2P2, CDAAs-T2P3, and CDAAs-T2P4. These curves manifest between class II and class IV isotherms with hysteresis loops conforming to the H3 classification, generally indicating a mesoporous structure nature in the pore distribution of CDAAs-T2P2, CDAAs-T2P3, and CDAAs-T2P4. Remarkably, the predominant pore sizes for CDAAs-T2P2, CDAAs-T2P3, and CDAAs-T2P4 cluster around 14, 10, and 12 nm, respectively. Compared to CDAAs-T2P3 and CDAAs-T2P4, the hysteresis loop of CDAAs-T2P2 is significantly smaller (Figure 4a). This phenomenon indicates that the number of large pores is higher in terms of CDAAs-T2P2, but the pores are still mostly mesopores. In addition, the specific surface areas for CDAAs-T2P2, CDAAs-T2P3, andCDAAs-T2P4 are 158.69, 112.01, and 147.24 m^2^/g, respectively. The results are consistent with the microstructure of the texture of aerogels prepared by FESEM, as in Figure 3.

### 2.2. Chemical Crosslinking Evidence

**Figure 5:**
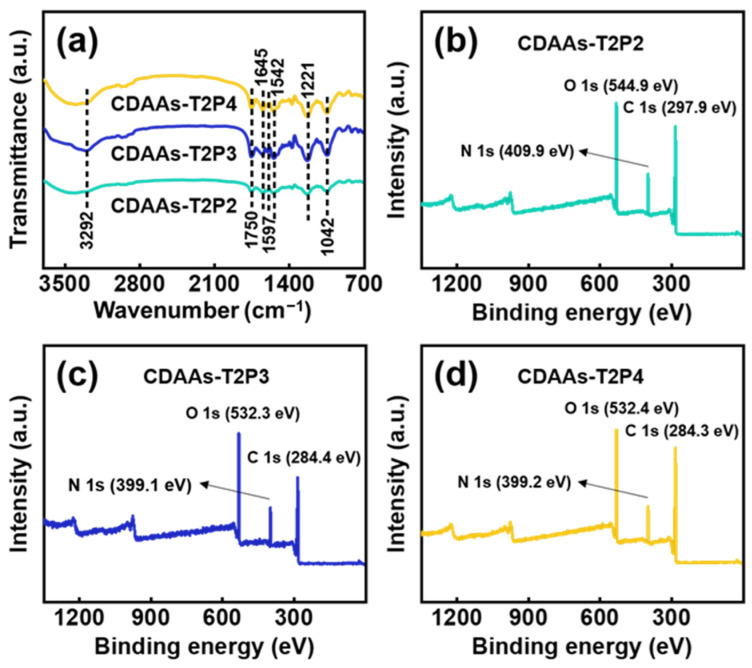
FTIR spectra of CDAAs (**a**) and XPS spectra of CDAAs: (**b**) CDAAs-T2P2, (**c**) CDAAs-T2P3, (**d**) CDAAs-T2P4.

FTIR spectroscopy is a popular technology today due to its unique combination of sensitivity, flexibility, specificity, and robustness. The chemical groups and compositions of CDAAs could be obtained by means of FTIR and XPS characterizations as provided in Figure 5. Based on the testing results, six distinct absorption peaks are evident as tested, corresponding to the wavenumbers of 3292, 1750, 1645, 1597, 1542, and 1221 cm^−1^, respectively. Among these, the peak at 3292 cm^−1^ signifies a N-H vibration, while those at 1750 and 1645 cm^−1^ denote C=O stretching and bending vibrations, respectively. Additionally, the peak at 1597 cm^−1^ signifies C=C stretching vibration within the benzene ring, and the peak at 1542 cm^−1^ represents a C-N stretching vibration and N-H bending vibration, whereas the peak at 1221 cm^−1^ indicates a C-O stretching vibration. It is noteworthy that no peaks corresponding to the cyanate ester group were observed in the FTIR spectra of CDAAs-T2P2, CDAAs-T2P3, and CDAAs-T2P4, indicating that the complete reactions for forming the networking skeletons of aerogels have indeed occurred between the cyanate groups and the urethane groups. The chemical crosslinking reactions responsible for the formation of cellulose diacetate aerogel are confirmed by these results.

As shown in Figure 5 and Figure S2, the binding energies of C1s and N1s further confirm the crosslinking reaction between TDI and CDA, which is consistent with the data of FTIR spectra of CDAAs. In the FTIR spectra of CDAAs-T2P3 and CDAAs-T2P4, the individual characteristic peaks exhibit heightened signals compared with the FTIR spectra of CDAAs-T2P2. This implies compelling evidence of an elevated crosslinking degree associated with the increased dosage of pyridine and the quantity of urethane involved. Therefore, the pyridine would increase the extent of the chemical reactions, resulting in a dense network of CDAAs.

**Figure 6:**
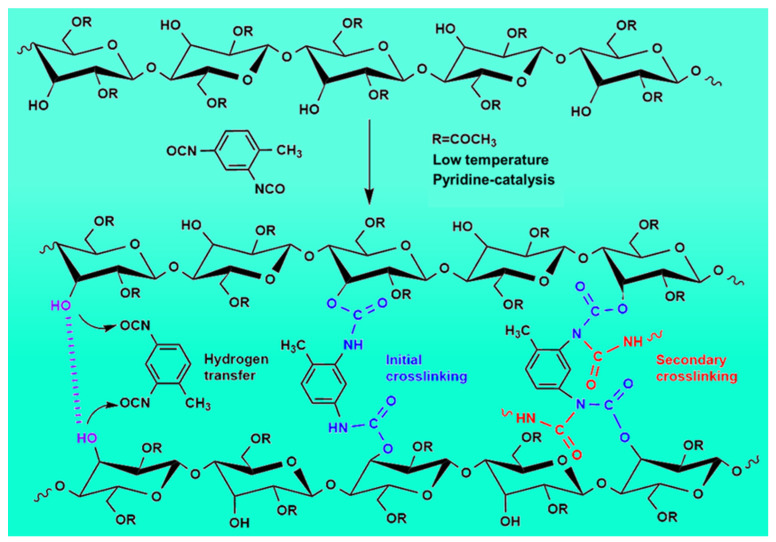
Chemical reaction process of CDAA formation.

Based on the evidence, Figure 6 illustrates the chemical reaction process of CDAAs. This process comprises two primary steps. Initially, in the condition of catalysis by pyridine, the hydroxyl groups (-OH) on the main chain of the cellulose diacetate molecules undergo a hydrogen-transfer reaction with the cyanate group (-N=C=O) on the crosslinking agent to form a urethane bond (-O-CONH-). Subsequently, the remaining crosslinker in the system starts a further hydrogen transfer reaction with the formed ammonia ester bonds to produce urethane carboxylate (-HNCON-), which facilitates the chemical crosslinking reactions of cellulose diacetate. Finally, the presence of hydrogen bonding interactions (between the hydroxyl groups (-OH) from the cellulose molecular chains) strengthens the cohesion among the molecular chains. The amalgamation of the chemical crosslinking process (between cellulose diacetate and the crosslinking agent) couples with robust hydrogen bonding interactions between the molecular chains, and further fosters the formation of a three-dimensional crosslinking network structure in CDAAs.

### 2.3. Thermal Insulation Performance

**Figure 7:**
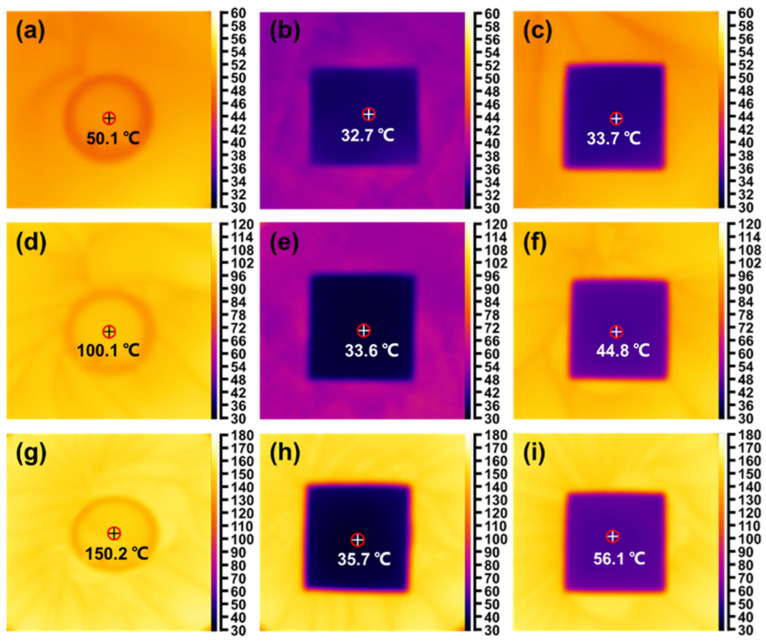
Monitoring temperature changes in the center of the cold surface of CDAAs-T2P4 on the heating plates at 50, 100, and 150 °C, respectively: CDAAs-T2P4 at (**a**–**c**) 50 °C, (**d**–**f**) 100 °C, and (**g**–**i**) 150 °C. Note: The red circle in the figure indicates the temperature measurement location.

Tests were carried out for the thermally insulating property of CDAAs-T2P4 in Figure 7. The man-made equipment was designed in more detail, such that the conical flask was placed in an oil bath and heated at a certain temperature. The sample of CDAAs-T2P4 was then positioned on the upper surface, and the contacting surface represented the hot side of CDAAs-T2P4. Conversely, the opposite side of the aerogel sample served as the cold surface. When the temperature was stabilized at around 50, 100, and 150 °C, the hot side (namely heat resource) of the conical flask was sealed with an anti-corrosive film. The purpose of employing a constant temperature via an oil bath was to establish stable temperature conditions for the upper surface of the conical flask, serving as the heat source. The sample was placed on the top side, and the surface that touched it was the hot side of CDAAs-T2P4. The other side of the aerogel was the cold surface.

Figure 7 shows the temperature changes of CDAAs-T2P4 at different temperatures of the hot surface (heat resource). As can be observed, when the hot surface temperatures of CDAAs-T2P4 were adjusted to 50.1, 100.1 and 150.2 °C respectively, the beginning temperatures matched the cold side temperatures of the specimens above of 32.7, 33.6, and 35.7 °C, respectively. After experiencing the heat flow balance, the cold side temperature was 33.7 °C at the condition of the hot surface temperature of 50.1 °C, the cold side temperature was 44.8 °C at the condition of the hot surface temperature of 100.1 °C, and the cold side temperature was only 56.1 °C at the hot surface temperature of 150.2 °C. Thus, CDAAs-T2P4 demonstrated a good thermal insulation property even at the high temperatures. This result may be owing to the nanoscale pore size and dense and homogenous network structure of cellulose acetate aerosols, which limits the gaseous conduction of heat within the pore and the lightweight network skeleton. This structure can eventually decrease the solid conductivity of heat to achieve superior thermal insulation.

### 2.4. Mechanical Property

**Figure 8:**
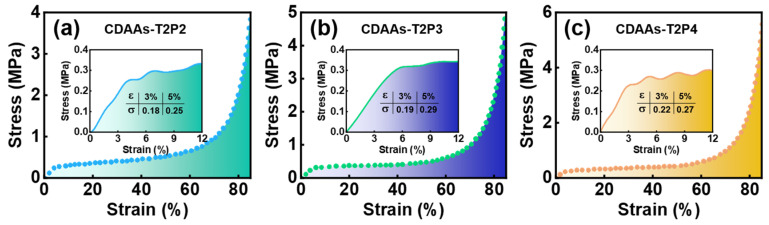
Compressive stress–strain curves of CDAAs: (**a**) CDAAs-T2P2, (**b**) CDAAs-T2P3, (**c**) CDAAs-T2P4.

To investigate the mechanical properties of the CDAAs-TP series, Figure 8 depicts the compressive stress–strain curves for CDAAs-T2P2, CDAAs-T2P3, and CDAAs-T2P4. The entire compression process can be separated into three zones: the elastic deformation zone, plastic deformation zone, and dense zone, indicating a linear relationship between stress and strain. The curve experiences a rapid increase in the low strain zone, with a linear correlation between stress and strain. Subsequently, the curve rises slowly and finally the stress increases exponentially with strain. At 3% strain, CDAAs-T2P2, CDAAs-T2P3, and CDAAs-T2P4 had compressive strengths of 0.18, 0.19, and 0.22 MPa respectively, exhibiting the excellent compressive properties of cellulose acetate aerogels. The compressive characteristics were improved with the increase in pyridine dosage, which was most likely owed to the increase in the amount of pyridine and the deeper chemical crosslinking. On the other hand, the robust networking skeleton resulted in an increase in the compressive strength, which is also well confirmed by its microstructure in Figure 3.

### 2.5. Thermal Stability Analysis

**Figure 9:**
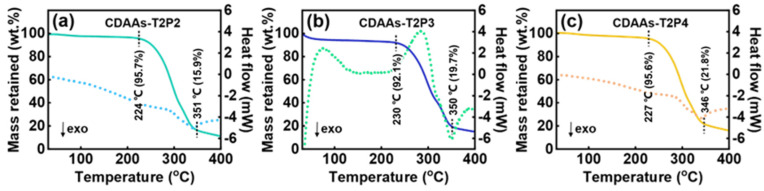
Thermal stability properties of CDAAs obtained by TG-DSC: (**a**) CDAAs-T2P2, (**b**) CDAAs-T2P3, (**c**) CDAAs-T2P4. Note: The solid line indicates the mass retention rate and the dashed line indicates the heat flow.

The thermal stability of the aerogels is a key aspect for practical applications of materials. Hence, we investigated the thermal stability of CDAAs in the condition of temperatures ranging from room temperature to 400 °C under the air atmosphere. By means of TG-DSC, the distinct stages of thermal decomposition are elucidated, as shown in Figure 9. The initial stages involve the evaporation of water from both as-prepared aerogels and residual raw materials, including any leftover solvent. Subsequently, a second stage ensues, marked by a sharp decline in the TG curve, indicating significant weight loss attributed to the decomposition of CDAAs. This phase is characterized by the rupture of interconnected molecular chains within the CDAAs’ structure. Finally, the curve exhibits a gradual descent towards equilibrium, attributable to residual carbon post-continual decomposition of CDAAs. Depending upon the data, the theoretical usable temperatures for CDAAs-T2P2, CDAAs-T2P3, and CDAAs-T2P4 were determined to be 224, 230, and 227 °C, respectively, representing their upper limits of thermal stability.

## 3. Conclusions

An aerogel is an ultra-light, porous material with a high specific surface area and high porosity (greater than 90%), which has been called “the wonder material of the 21st century”. A cellulose aerogel is primarily composed of cellulose with environment-friendly properties, with a series of excellent characteristics such as easy surface modification, high porosity, high specific surface area, low density, and three-dimensional interconnectivity. Cellulose aerogels are also renewable and biodegradable and are known as “the third-generation aerogel”. The hierarchical structure of cellulose, from polymers to nano/microfibers, facilitates a range of material synthesis across scales, increasing its applicability.

In this study, cellulose diacetate aerogels were successfully prepared with low drying shrinkage, promising thermal insulation, and good mechanical strength, and the crosslinker and catalyst could effectively modulate the as-prepared aerogel pores, especially for the large pores transformed to the small one. Moreover, tert-butanol as an exchange solvent can contribute significantly to the formation of CDAAs with a networking structure. Minimizing capillary pressure and ensuring a strong structure construction is possible with an exchange solvent that has a low surface tension.

The aerogels exhibited low drying shrinkage (as low as 1.4%) and were lightweight (0.069 g cm^−3^). The CDAAs synthesized also displayed excellent mechanical properties (0.22 MPa at 3% strain) and low thermal conductivity (as low as to 0.021 W m^−1^ K^−1^ at room temperature and normal pressure), whose cold surface temperatures were only 56.1 °C when the hot surface was 150 °C after the balanced heat reflux. Therefore, cellulose-derived aerogels can be used to prepare biomass aerogels, which are used for the high-performance environmental protection insulation of energy-efficient buildings and the reduction of energy consumption. The above evidence in this work confirms indeed the excellent thermal insulation and superior compressive properties of CDAAs. This research may offer a kind of reference for constructing high-performance biomass-based aerogels including cellulose-derived aerogels in the future.

## 4. Materials and Methods

### 4.1. Materials

Cellulose diacetate (CDA, 39.8 wt.% acetyl content), N-methyl pyrrolidone (NMP, AR), pyridine (Py, AR), tert-butanol (TBA, AR), and 2,4-toluene diisocyanate (TDI) were obtained from Shanghai Aladdin Biochemical Technology Co., Ltd. (Shanghai, China). The reagents mentioned above were used without further purification in this experiment.

### 4.2. Preparation of CDAAs

CDA, TDI, Py, and NMP were used as a raw material, crosslinker, catalyst, and solvent, respectively. CDA was mixed with NMP at a low temperature (0 °C) and stirred vigorously for about 1 h until a clarified and homogeneous solution was formed. After that, the pyridine was added slowly dropwise to the above solution and synchronously stirred, and then TDI was added and stirred for 15–30 min. When the bubbles started to appear in the mixed solution under the vigorous stirring, then the solution was poured into a cylindrical mold and sealed with plastic wrap. The solution was placed in an oven at 75 °C for six hours to obtain the CDA gel. The CDA gel was immersed in TBA solution for aging and solvent exchange to remove the NMP solvent inside the gel. In the end, the CDAAs were obtained by supercritical drying of the CDA gel at 60 °C and 17.5 MPa.

Experiments were carried out with nine different reactant ratios as shown in Table S1. Due to the high concentration of CDA, it was difficult to disperse it uniformly in NMP solvent due to the high viscosity of the mixed solution. Thus, the CDA solution in NMP solvent was moderately stirred at a particular revolution to ultimately achieve the corresponding solution. During mixing with the crosslinker, the mixed solution tended to have many bubbles, resulting in an inconsistent internal structure of the gel in some regions. In the consideration of a single-variable method in this work, the other factors were designed for the solvent volume of 300 mL, the mass of CDA for 9 g, and the dosage of Py of 9 mL.

According to the experimental methods above and the fact that the vital factor affecting the outcome was the dosage of TDI, the categorization of tests was abbreviated to CDAAs-T1, CDAAs-T2, CDAAs-T3, and CDAAs-T4, with unchanged conditions of the other factors (detailed dosages for the corresponding factors are presented in Table S1). Notably, the comprehensive properties exhibited by CDAAs-T2 in Figure S1 were exemplary in all the tests, demonstrating no discernible bending or fragmentation from a macro point of view. Consequently, depending upon the optimal CDAAs-T2 group demonstrated by the tests above, the catalyst dosage was found as the principal influencing parameter (with dosages ranging from 8.0 to 10.0 mL). The resultant aerogels were denoted as CDAAs-T2P1, CDAAs-T2P2, CDAAs-T2P3, CDAAs-T2P4, and CDAAs-T2P5, respectively.

### 4.3. Characterization Measurements

The surface micromorphology was observed using a field emission scanning electron microscope (FESEM, S4800, Hitachi Ltd., Tokyo, Japan) with an accelerating voltage setting of 3 kV and a working distance of 5.1 mm. Nitrogen adsorption–desorption experiments were performed using an Autosorb IQ analyzer (ASAP 2020 HD88 Mike Instrument, Atlanta, GA, USA). The samples were degassed at 90 °C for 12 h prior to the test. The total pore volume and pore diameter were measured by the Barrett–Joyner–Halenda (BJH) analysis. Chemical composition (in the range of 400–4000 cm^−1^ with the weight ratio of 1:99 for CDAAs to KBr) was determined using a Fourier transform infrared spectrometer (FTIR, Nicolet iS5, Thermo Fisher Scientific, Waltham, MA, USA) and X-ray photoelectron spectroscopy (XPS, Thermo Scientific K-Alpha, Waltham, MA, USA). The dense layer on the sample surface was ground off and the thermal conductivity of the samples was tested at 25 °C using a thermal conductivity meter (TC3000E, Xi’an Xiaxi Electronic Technology Co., Ltd., Shaanxi, China). The compressive stress–strain curves were evaluated using a testing machine (XBD4204, Zhejiang Sanshi Yongheng Technology Co., Ltd., Zhejiang, China) with a load cell of 20 kN and a compressive rate of 0.5 mm/min. The thermal insulation property of CDAAs was tested by self-assembled equipment including a thermostatic water bath (DF-101S, Henan Baize Instrument Co., Ltd., Henan, China) and infrared thermal imager (Fluke Tis 60+, Fluke Testing Instruments (Shanghai) Co., Ltd., Shanghai, China) with Sony camera recorder (α7 II, Sony (China) Co., Ltd., Beijing, China). Thermogravimetry analysis (TG, Q500, TA Instrument Co., New Castle, DE, USA) and differential scanning calorimetry (DSC, Q200, TA Instrument Co., New Castle, DE, USA) were also performed. In the air atmosphere with a heating rate of 10 °C/min, the analysis temperature was adjusted to a range of 30 to 500 °C.

## Data Availability

Data are available from the authors. Samples of the compounds are available from the authors.

## Supplementary Materials

The following supporting information can be downloaded at: https://www.mdpi.com/article/10.3390/gels10030210/s1, Figure S1: Photographs of the corresponding sol, initial gel, final gel and aerogel in preparing CDAAs processes; Figure S2: XPS spectra of CDAAs; Table S1: Chemical composition and corresponding properties of CDAAs.

## References

[1] Zhang S., He J., Xiong S., Xiao Q., Xiao Y., Ding F., Ji H., Yang Z., Li Z. (2021). Construction and nanostructure of chitosan/nanocellulose hybrid aerogels. Biomacromolecules.

[2] Yao C., Yin X., Yu Y., Cai Z., Wang X. (2017). Chemically functionalized natural cellulose materials for effective triboelectric nanogenerator development. Adv. Funct. Mater..

[3] Chang X.X., Mubarak N.M., Mazari S.A., Jatoi A.S., Ahmad A., Khalid M., Walvekar R., Abdullah E.C., Karri R.R., Siddiqui M.T.H. (2021). A review on the properties and applications of chitosan, cellulose and deep eutectic solvent in green chemistry. J. Ind. Eng. Chem..

[4] Tu H., Zhu M., Duan B., Zhang L. (2021). Recent progress in high-strength and robust regenerated cellulose materials. Adv. Mater..

[5] Wang X., Pang Z., Chen C., Xia Q., Zhou Y., Ray S.J.R.W.U., Gan W., Li C., Chen G., Foster B. (2020). All-natural, degradable, rolled-up straws based on cellulose micro-and nano-hybrid fibers. Adv. Funct. Mater..

[6] Shchipunov Y., Postnova I. (2018). Cellulose mineralization as a route for novel functional materials. Adv. Funct. Mater..

[7] Xiong S., Hu Y., Zhang S., Xiao Y., Li Z. (2021). Constructing Cellulose Diacetate Aerogels with Pearl-Necklace-like Skeleton Networking Structure. Gels.

[8] Zhou S., Nyholm L., Strømme M., Wang Z. (2019). Cladophora cellulose: Unique biopolymer nanofibrils for emerging energy, environmental, and life science applications. Acc. Chem. Res..

[9] Hees T., Zhong F., Rudolph T., Walther A., Mülhaupt R. (2017). Nanocellulose aerogels for supporting iron catalysts and in situ formation of polyethylene nanocomposites. Adv. Funct. Mater..

[10] Rehman A., Nazir G., Rhee K.Y., Park S.-J. (2021). A rational design of cellulose-based heteroatom-doped porous carbons: Promising contenders for CO_2_ adsorption and separation. Chem. Eng. J..

[11] Al Abdallah H., Abu-Jdayil B., Iqbal M.Z. (2022). Improvement of mechanical properties and water resistance of bio-based thermal insulation material via silane treatment. J. Clean. Prod..

[12] Qian H., Liu J., Wang X., Pei W., Fu C., Ma M., Huang C. (2022). The state-of-the-art application of functional bacterial cellulose-based materials in biomedical fields. Carbohydr. Polym..

[13] Kim J.-H., Lee D., Lee Y.-H., Chen W., Lee S.-Y. (2019). Nanocellulose for energy storage systems: Beyond the limits of synthetic materials. Adv. Mater..

[14] AscanioVillabona J., Romero B.E.T., Duran M.A., Lengerke O., Betancur L. (2023). Evaluation of the thermal performance of housing envelopes as passive cooling systems. Sustain. Eng. Innov..

[15] Jain R., Bakare Y.B., Pattanaik B., Alaric J.S., Balam S.K., Ayele T.B., Nalagandla R. (2023). Optimization of energy consumption in smart homes using firefly algorithm and deep neural networks. Sustain. Eng. Innov..

[16] Adrian M., Purnomo E.P., Enrici A., Khairunnisa T. (2023). Energy transition towards renewable energy in Indonesia. Herit. Sustain. Dev..

[17] Hees T., Zhong F., Rudolph T., Walther A., Mülhaupt R. (2020). PLA coating improves the performance of renewable adsorbent pads based on cellulosic aerogels from aquatic waste biomass. Chem. Eng. J..

[18] Zhou J., Zhang R., Xu R., Li Y., Tian W., Gao M., Wang M., Li D., Liang X., Xie L. (2022). Super-Assembled Hierarchical Cellulose Aerogel-Gelatin Solid Electrolyte for Implantable and Biodegradable Zinc Ion Battery. Adv. Funct. Mater..

[19] Zhang Y.C.L., Yang Y., Pang B., Xu W., Duan G., Jiang S., Zhang K. (2021). Recent progress on nanocellulose aerogels: Preparation, modification, composite fabrication, applications. Adv. Mater..

[20] Gong C., Ni J.-P., Tian C., Su Z.-H. (2021). Research in porous structure of cellulose aerogel made from cellulose nanofibrils. Int. J. Biol. Macromol..

[21] Peng Q., Lu Y., Li Z., Zhang J., Zong L. (2022). Biomimetic, hierarchical-ordered cellulose nanoclaw hybrid aerogel with high strength and thermal insulation. Carbohydr. Polym..

[22] Chen G., Hong F.F., Yuan J., Li L., Fang M., Wei W., Wang X., Wei Y. (2022). Super solvent of cellulose with extra high solubility for tunable cellulose structure with versatile application. Carbohydr. Polym..

[23] Yin S., Zhang X., Hu G., Huang T., Yu H., Yu B., Zhu M. (2022). In situ crosslinking of mechanically robust waterproof and moisture permeable cellulose diacetate nanofiber aerogels for warm clothing. Chem. Eng. J..

[24] Guo L., Chen Z., Lyu S., Fu F., Wang S. (2018). Highly flexible cross-linked cellulose nanofibril sponge-like aerogels with improved mechanical property and enhanced flame retardancy. Carbohydr. Polym..

[25] Jiang F., Hsieh Y.-L. (2017). Cellulose nanofibril aerogels: Synergistic improvement of hydrophobicity, strength, and thermal stability via cross-linking with diisocyanate. ACS Appl. Mater. Interfaces.

[26] Jiang F., Hsieh Y.-L. (2021). Crosslinking polydopamine/cellulose nanofibril composite aerogels by metal coordination bonds for significantly improved thermal stability, flame resistance, and thermal insulation properties. Cellulose.

[27] Zhou T., Cheng X., Pan Y., Li C., Gong L. (2019). Mechanical performance and thermal stability of polyvinyl alcohol-cellulose aerogels by freeze drying. Cellulose.

[28] Zhang S., Huang X., Feng J., Qi F., Dianyu E., Jiang Y., Li L., Xiong S., Fen J. (2020). Structure, compression and thermally insulating properties of cellulose diacetate-based aerogels. Mater. Des..

[29] Zhang S., Wang Z., Hu Y., Ji H., Xiao Y., Wang J., Ding G.X.F. (2022). Ambient Pressure Drying to Construct Cellulose Acetate/Benzoxazine Hybrid Aerogels with Flame Retardancy, Excellent Thermal Stability, and Superior Mechanical Strength Resistance to Cryogenic Temperature. Biomacromolecules.

[30] Martínez-Lázaro A., Ramírez-Montoya L.A., Ledesma-García J., Montes-Morán M.A., Gurrola M.P., Menéndez J.A., Arenillas A., Arriaga L.G. (2022). Facile Synthesis of Unsupported Pd Aerogel for High Performance Formic Acid Microfluidic Fuel Cell. Materials.

