# Supplementary material for: Cellulose Diacetate Aerogels with Low Drying Shrinkage, High-Efficient Thermal Insulation, and Superior Mechanical Strength
Source: Gels. 2024 Mar 21;10(3):210. doi: 10.3390/gels10030210 (PMC10970052; doi:10.3390/gels10030210)
Supplement: Supplementary file 1 [file gels-10-00210-s001.zip › gels-2888616-supplementary.pdf]

Supporting Information

Cellulose Diacetate Aerogels with Low Drying Shrinkage, High-Efficient Thermal Insulation, Superior Mechanical Strength

Sizhao Zhang [1,2,3,*], Kunming Lu [1], Yangbiao Hu [1], Guangyu Xu [1], Jing Wang [1], Yanrong Liao [1] and Shuai Yu [1]

1 Polymer Aerogels Research Center, Jiangxi University of Science and Technology, Nanchang 330013, China
2 Science and Technology on Advanced Ceramic Fibers and Composites Laboratory, National University of Defense Technology, Changsha 410073, China
3 Postdoctoral Research Station on Mechanics, College of Aerospace Science and Engineering, National University of Defense Technology, Changsha 410073, China
* Correspondence: zhangsz@jxust.edu.cn

Contents of Figures and Tables

Figure S1: Photographs of the corresponding sol, initial gel, final gel and aerogel in preparing CDAAs processes. The respective stages for (a, e, i, m) CDAAs-T1, (b, f, j, n) CDAAs-T2, (c, g, k, o) CDAAs-T3, (d, h, l, p) CDAAs-T4.

Figure S2: XPS spectra of CDAAs: (a-c) CDAAs-T2P2, (d-f) CDAAs-T2P3, (g-i) CDAAs-T2P4.

Table S1: Chemical composition and corresponding properties of CDAAs.

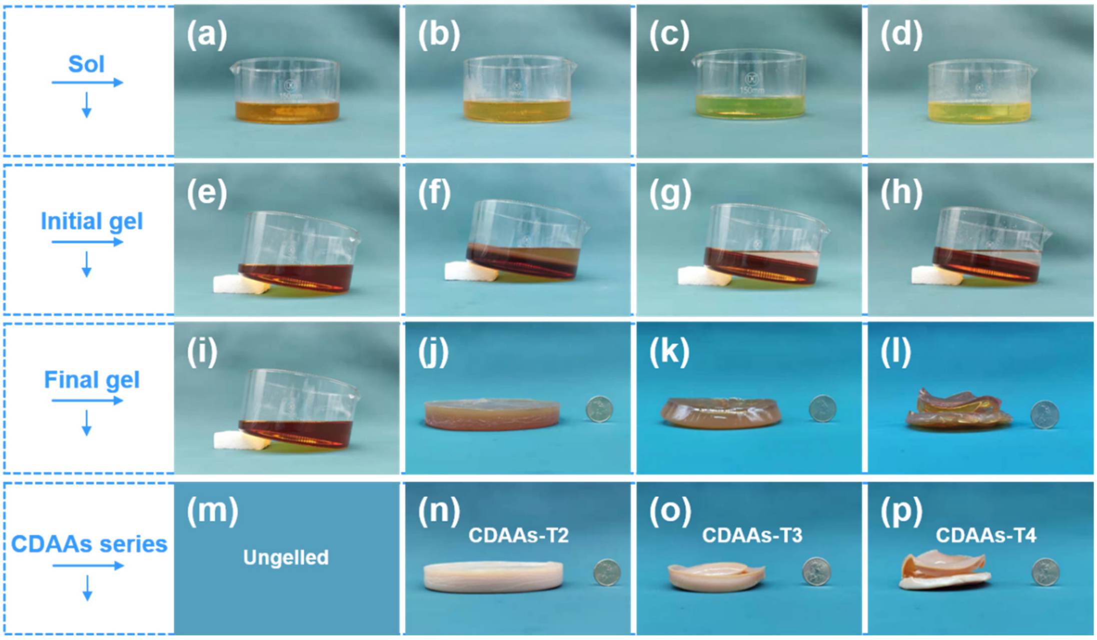

Figure S1. Photographs of the corresponding sol, initial gel, final gel and aerogel in preparing CDAAs processes. The respective stages for (a, e, i, m) CDAAs-T1, (b, f, j, n) CDAAs-T2, (c, g, k, o) CDAAs-T3, (d, h, l, p) CDAAs-T4.

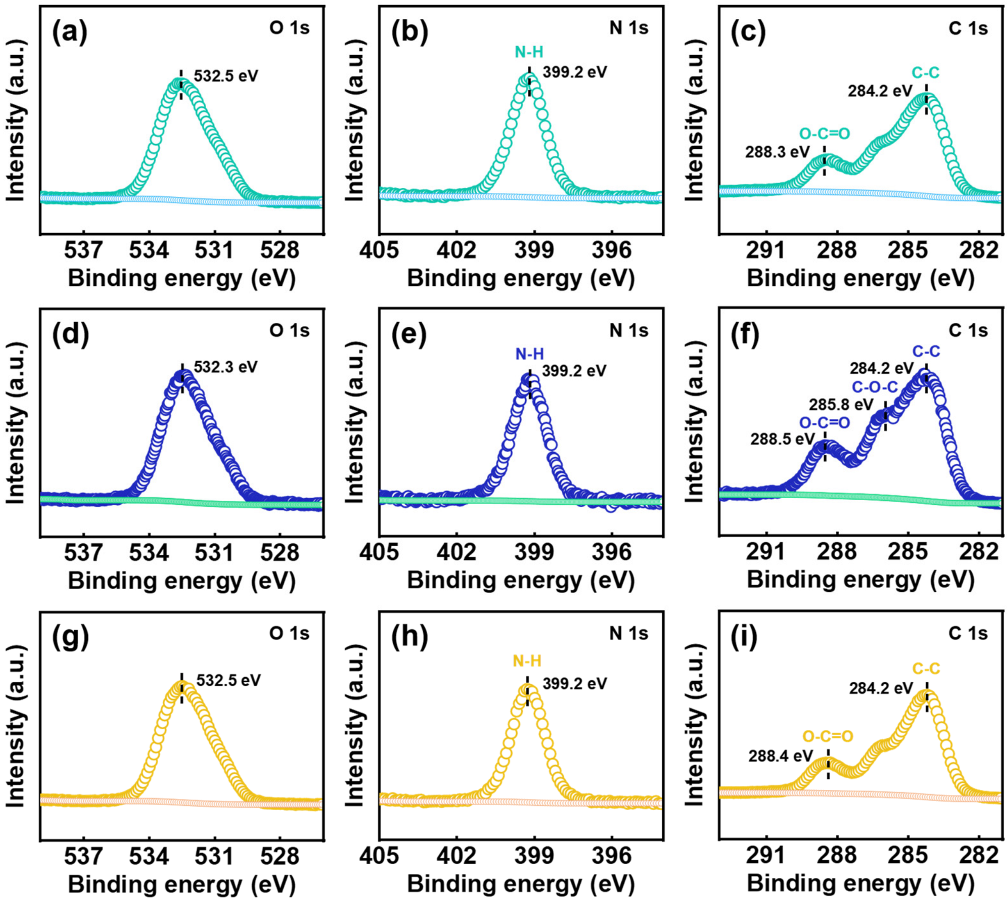

Figure S2. XPS spectra of CDAAs: (a-c) CDAAs-T2P2, (d-f) CDAAs-T2P3, (g-i) CDAAs-T2P4.

Table S1. Chemical composition and corresponding properties of CDAAs. Note: the preparation system all controlled in the sum volume of 300 mL; TC refers to thermal conductivity.

Specimens	CDA (g)	TDI (g)	Py (mL)	ρ (g cm^{-3})	TC (W m^{-1} K^{-1})
CDAAs-T1	9	9.44	9.0	—	—
CDAAs-T2	9	10.20	9.0	0.073	0.021
CDAAs-T3	9	10.98	9.0	0.391	—
CDAAs-T4	9	11.75	9.0	0.405	—
CDAAs-T2P1	9	10.20	8.0	0.209	—
CDAAs-T2P2	9	10.20	8.5	0.069	0.025
CDAAs-T2P3	9	10.20	9.0	0.073	0.021
CDAAs-T2P4	9	10.20	9.5	0.079	0.023
CDAAs-T2P5	9	10.20	10.0	0.223	0.030
